# Impact of Extracorporeal Membrane Oxygenation in an Infant Treated with Vancomycin: A Case Report

**DOI:** 10.3390/ijerph20031839

**Published:** 2023-01-19

**Authors:** Chihiro Shiraishi, Hideo Kato, Hiroshi Imai, Takuya Iwamoto

**Affiliations:** 1 Department of Pharmacy, Mie University Hospital, Tsu 514-8507, Japan; 2 Department of Clinical Pharmaceutics, Division of Clinical Medical Science, Mie University Graduate School of Medicine, Tsu 514-8507, Japan; 3 Emergency and Critical Care Center, Mie University Hospital, Tsu 514-8507, Japan

**Keywords:** infant, vancomycin, pharmacokinetic, pharmacodynamic, extracorporeal membrane oxygenation

## Abstract

Vancomycin is a glycopeptide antibiotic used for prophylaxis and treatment of infections caused by methicillin-resistant Staphylococcus aureus. Although major organ sizes and functions mature during infancy, pharmacokinetic studies, especially those focused on infants, are limited. Changes in extracorporeal membrane oxygenation-related drug disposition largely contribute to changes in pharmacokinetics. Here, pharmacokinetic profiles of vancomycin in an infant receiving extracorporeal membrane oxygenation therapy are presented. A two-month-old Japanese infant with moderately decreased renal function was started on 12.0 mg/kg vancomycin every 8 h from day X for prophylaxis of pneumonia during extracorporeal membrane oxygenation therapy. As the trough concentration of vancomycin observed on day X+3 was 27.1 μg/mL, vancomycin was then discontinued. The trough concentration decreased to 18.6 μg/mL 24 h after discontinuation, and 9.0 mg/kg vancomycin every 12 h was restarted from day X+5. On day X+6, the trough concentration increased to 36.1 μg/mL, and vancomycin therapy was again discontinued. On day X+7, the trough concentration decreased to 22.4 μg/mL. The pharmacokinetic profiles of vancomycin based on first-order conditional estimation in this infant were as follows: plasma clearance = 0.053 L/kg/hour, distribution volume = 2.19 L/kg, and half-life = 29.5 h. This research reported the prolonged half-life of vancomycin during extracorporeal membrane oxygenation in infants with moderately decreased renal function.

## 1. Introduction

Vancomycin (VCM) is a glycopeptide antibiotic used in the treatment of resistant Gram-positive infections, especially those caused by methicillin-resistant *Staphylococcus aureus* [1]. The total clearance (CL) of VCM is related to renal function because it is eliminated by glomerular filtration [2]. Moreover, the distribution volume (*Vd*) is generally affected by body weight. The Food and Drug Administration has proposed classifying the pediatric population as neonates (birth to 1 month of life), infants (1–24 months), children (2–11 years), and adolescents (12–18 years) based on the complex changes and the anatomical, biochemical, and physiological differences related to age [3]. Pediatric patients have a very wide range of physical development processes from newborns to adolescents, which have different effects on the CL and *Vd* of VCM at each growth process in the pediatric population [4]. Therefore, understanding the pharmacokinetic (PK) profile of VCM is critical for establishing an optimal dosing regimen for VCM.

Extracorporeal membrane oxygenation (ECMO) is a cardiopulmonary support system used in patients with potentially reversible respiratory or cardiac failure and works by increasing the circulating blood volume and transiently altering renal function [5]. ECMO increases the risk of serious infections because of its invasiveness, central catheter-related bacteremia, multiorgan failure, and immunosuppression. Therefore, antibiotics are frequently used for both the prophylaxis and treatment of patients treated with ECMO [6]. However, since ECMO increases circulating blood volume and transiently alters renal function, it may affect the PK profiles of antibiotics in patients treated with ECMO [7].

To date, several studies have investigated the effects of ECMO on the PK of VCM in the pediatric population [8,9]. However, no study has focused on infants. Here, the PK profile of VCM in an infant who underwent ECMO therapy is presented.

## 2. Case Report

A two-month (65 days)-old Japanese male infant (gestational age: 41 weeks, birth weight: 3178 g, birth height: 50.0 cm, and Apgar score: 8–9) had a history of circulatory insufficiency of ventricular septal defect and interruption of the aortic arch. After Norwood and right ventricle-to-pulmonary artery conduit surgery (day X−4), lactic acidosis (lactate: 20 mmol/L, pH: 7.11) was observed. In addition, a decline in blood pressure (101/30 mmHg) and saturation of percutaneous oxygen level (40–50%) were observed. Since the patient was in a complicated state with poor ventilation and severe neonatal circulatory failure, intensive care including Veno-Arterial (VA) ECMO therapy (priming volume: 300 mL; BIOCUBE C2000P, HP2 03248702H0, HP2 03248706H0, NIPRO) were started on day X−4. Since sepsis, disseminated intravascular coagulation, and convulsions were observed, administration of 60 mg meropenem (13.6 mg/kg, body weight: 4380 g) every 8 h (h) were started on day X−4 as empiric therapy. On day X, increased white blood cell (WBC) count and C-reactive protein (CRP) levels were observed (WBC: 9.5 × 10^3^/μL, normal range: 3.3–8.6 × 10^3^/μL; CRP: 6.2 mg/dL, normal range: <0.1 mg/dL). For a prophylaxis of pneumonia, VCM (0.5 g vancomycin hydrochloride for i.v. infusion, meiji) was administered as an intravenous (IV) infusion over 60 min immediately after collecting one set of suction phlegm cultures. The initial VCM dose was 55 mg (12.5 mg/kg) administered every 8 h (5:00, 13:00, and 21:00). The serum trough concentrations were measured using the PETINIA method (Dimension Xpand, Siemens). A trough sample was obtained 30 min before VCM administration. Since serum trough concentrations of VCM were 27.1 μg/mL at 13:00 on day X+3, VCM administration at 21:00 on day X+3, 5:00 and 13:00 on day X+4 was discontinued. At 24 h after the first trough measurement, the serum trough concentration of VCM decreased to 18.6 μg/mL at 13:00 on day X+4, and the VCM dose was restarted from day X+5 at 40 mg (9.0 mg/kg) every 12 h. After conducting a clamp test for ECMO weaning on days X+5 and X+6, an elevated WBC count (16.9 × 10^3^/µL) and an increased CRP level (22.7 mg/dL) were noted. Despite reducing the VCM dose from day X+4, the serum concentration of VCM increased to 36.1 μg/mL on day X+6, and VCM was again discontinued. The next day (day X+7), the serum trough concentration decreased to 22.4 μg/mL. An increase in serum creatinine (SCr) and blood urea nitrogen (BUN), and a decrease in renal function was observed from day X to day X+7 (day X vs. day X+7: SCr, 0.5 mg/dL vs. 0.7 mg/dL; BUN, 45.7 mg/dL vs. 91.6 mg/dL; glomerular filtration rate [GFR], 55.4 mL/min/1.73 m^2^ vs. 39.5 mL/min/1.73 m^2^). GFR was calculated as follows: GFR (mL/min/1.73 m^2^) = 0.45 × height (cm)/SCr (mg/dL) [10,11]. Based on the extrapolation of the criteria for adults, moderately decreased renal function was observed after ECMO therapy and administration of VCM [12]. VCM was administered to day X+14. The height of the infant at VCM administration was 61.5 cm. Suction phlegm and blood cultures collected on days X, X+4, X+6, and X+13 were all negative. The summary of laboratory variables in this infant case are provided in Table 1.

## 3. Pharmacokinetic Profile

The PK parameters based on observed serum VCM concentrations on day X+3 (27.1 μg/mL) and day X+4 (18.6 μg/mL) were calculated using first-order conditional estimation because VCM administrations were skipped during these periods. The *t*_1/2_ and *k* based on observed serum VCM concentrations of 27.1 μg/mL (*C*_0_) and 18.6 μg/mL (*C*) were calculated using the equation of *C* = *C*_0_ × *e*^−*kt*^. The *t*_1/2_ and *k* were 29.5 h and 0.024 h^−1^, respectively. The *Vd* was calculated to be 2.19 L/kg using the equation of $C_{trough}=\frac{\frac{\left(S\right)\left(F\right)\left(dose\right)}{Vd}}{1-e^{-k\tau }}\times e^{-k\tau }$ [13], where *S* is the salt index (1.0), *F* is bioavailability of VCM (1.0), and *τ* is the duration of VCM administration (8 h). The CL was calculated to be 0.053 L/kg/h using the equation of CL = *k* × *Vd*. *C* is the subsequent VCM concentration, *C*_0_ is the initial VCM concentration, *e* is the base of the natural logarithm, *k* is the elimination rate constant, *t* is the time interval between the initial and subsequent VCM concentrations.

## 4. Discussion

To date, one case report and two PK studies investigating the optimal dosage of VCM in pediatric patients treated with ECMO have been published [6,14,15]; however, these studies have focused on neonates from birth to one-month-old in age. Here, an increase in VCM concentration and prolongation of VCM elimination is reported in an infant patient receiving VCM during ECMO therapy, which was due to decreased renal function and increased *Vd*. These findings indicate the importance of extended dosing intervals and close monitoring of VCM concentrations in infants treated with ECMO. Regarding PK study of neonates receiving VCM during ECMO therapy, Amaker et al. reported the PK profiles of VCM in 12 neonates undergoing ECMO (mean SCr = 1.2 ± 0.5 mg/dL; severely decreased renal function), and the results were compared with previously published data. They showed increased *Vd* and *t*_1/2_ and decreased plasma CL levels [16]. In addition, Buck compared the PK profiles of VCM in neonates receiving ECMO therapy (n = 15; mean SCr = 0.8 ± 0.1 mg/dL; moderately decreased renal function) with neonates who did not receive ECMO therapy (n = 15; mean SCr = 0.6 ± 0.2 mg/dL; moderately decreased renal function) using steady state peak and trough concentrations, and the study found relatively larger *Vd* and lower plasma CL and a significantly prolonged *t*_1/2_ in neonates receiving ECMO therapy [6]. Although VCM CL is strongly associated with SCr [16], these two reports demonstrated similar PK changes. Therefore, the VCM profiles in infants may be similar to those in neonates undergoing ECMO therapy.

Body composition and organ maturation in the pediatric population develop during the first two years of age [17,18]. Total body water, expressed as percentage of body weight, decreases with age, from approximately 80% in neonates to 60% by one year of age; these changes could affect the *Vd* of VCM [19]. The volume of priming fluid in the ECMO system is likely to have a more profound effect on *Vd* of VCM in neonates and infants who have a smaller circulating blood volume compared with older children and adults because of a larger priming/blood volume ratio [20]. In this infant, the blood volume is estimated to be approximately 374 mL (85 mL/kg) [21]. Combining the blood and priming volume of ECMO (300 mL), the circulating blood volume is expected to more than double during ECMO therapy [21,22]. Regarding other factors, physiologic changes related to ECMO support and critical illness can also affect *Vd*. Exposure to the ECMO circuit results in an inflammatory response, which often results in increased Vd by capillary leak and edema. In addition, the renin-angiotensin system in the kidney can be upregulated, possibly related to non-pulsatile blood flow seen in VA EMCO. Upregulation of the renin-angiotensin system alters handling of fluids and can change the ratio of fluids in the body fluid compartments [22,23].

Administering VCM 10–20 mg/kg every 8–48 h depending on postmenstrual age, weight, and SCr has commonly been recommended for infants to achieve an area under the blood concentration-time curve (AUC) of 400 mg × h/L [24,25]. However, these studies enrolled infants who did not receive renal replacement therapy or ECMO. A PK study in pediatric patients treated with ECMO showed prolonged *t*_1/2_ of VCM in neonates receiving ECMO therapy, indicating that a dose of 20 mg/kg once daily is recommended for VCM [16]. Therefore, once daily VCM administration may be an adequate regimen for infants during ECMO therapy. However, there are limitations to the proposal of an adequate dosage of VCM in this manuscript. Gentamicin pharmacokinetics have also been well described in infants on ECMO. However, results are not consistent. When compared with infants without ECMO, one study reported that infants on ECMO had increased *Vd* and decreased total CL [26]. Two other studies reported similar *Vd* with decreased total CL [27,28]. Another study reported no difference in *Vd* or total CL [29]. Although there is not a robust consensus, previous studies tended to recommend a standard dose with a longer dosing interval to account for decreased total CL seen in infants on ECMO.

Nephrotoxicity is a major adverse event associated with VCM. The incidence of nephrotoxicity further increases in pediatric patients who achieve a VCM trough concentration of >15 mg/L [30] and are concurrently administered nephrotoxic agents (that is, loop diuretics, vasopressors, angiotensin-converting enzyme inhibitors, and non-steroidal anti-inflammatory) [31]. In this infant, furosemide was concomitantly administered with VCM during ECMO therapy and the GFR decreased. Therefore, high trough concentrations of VCM and concomitant use of nephrotoxic agents might also influence the incidence of nephrotoxicity in infants.

This manuscript is a case report on the PK profile of VCM in infants and provides manuscript limitations to be addressed in the future. First, this is a case report of only one infant patient with very few available concentrations. Moreover, there is no PK model of VCM for only infant patients. Therefore, further study is needed to rigorously estimate the PK parameters of infant patients with ECMO therapy. Second, the AUC as well as trough concentrations are considered for any dosage recommendation to minimize treatment failure in the neonate population [32]. However, the influence of AUC on bactericidal effects has not been established in the infant population, which needs to be addressed in future studies.

## 5. Conclusions

In conclusion, this manuscript reported the PK profile of VCM during ECMO in an infant with moderately impaired renal function. Our case report indicates that a once-daily dose of VCM might be appropriate due to prolonged *t*_1/2_, and further studies to determine the adequate dosage of VCM in infants receiving ECMO therapy are needed. Moreover, early and frequent monitoring of VCM concentrations is important in infants receiving both nephrotoxic agents and ECMO therapy. This report is expected to facilitate the further development of VCM therapy for infants receiving ECMO therapy.

## Figures and Tables

**Table 1:** Laboratory variables during pre-ECMO (day X−4 to day X−1), ECMO (day X to day X+10), and post-ECMO (day X+11 to day X+14).

	X−4	X−3	X	X+1	X+2	X+3	X+4	X+5	X+6	X+7	X+8	X+9	X+10	X+11	X+12	X+13	X+14
Plasma free Hb, g/L		0.08	0.08	0.06	0.06	0.09	0.13	0.03	0.05	0.09	0.09						
SCr, mg/dL	0.2	0.4	0.5	0.5	0.3	0.3	0.3	0.5	0.6	0.7	0.6	0.6	0.6	0.4	0.3	0.3	0.3
GFR, mL/min/1.73 m^2^	138.4	69.2	55.4	55.4	92.3	92.3	92.3	55.4	46.1	39.5	46.1	46.1	46.1	69.2	92.3	92.3	92.3
BUN, mg/dL	10.9	11.2	45.7	70.8	67.3	72.4	78.2	85.3	90.2	91.6	104.9	109.3	99.3	107.8	88.7	80.0	75.0
AST, IU	29	55	122	115	79	73	74	43	86	100		151		57	51	49	75
ALT, IU	25	16	34	26	23	19	20	13	21	30	43	53	50	56	57	58	58
Thrombocytes, 10^3^/µL	276	194	53	31	77	120	121	197	167	280	172	64	51	121	119	106	107
Total bilirubin, mg/dL	0.2	2.2	2.4	3.9	2.3	2.8	2.8	1.5	2.5	4.4	4.7	4.6	4.2	3.7	2.9	2.0	1.6
Serum potassium, mmol/L	4.5	4.7	4.9	3.8	3.6	3.7	3.5	4.1	4.6	5.0	5.0	4.3	4.4	3.9	4.3	3.2	3.2
CRP, mg/dL	1.1	1.1	6.2	3.0	2.0	2.7	5.8	6.6	22.7	25.8	21.8	12.2	15.3	11.3	8.0	6.4	5.7
PCT, ng/mL		2.5		1.5		0.4				>75.0	>75.0	73.3	71.8	42.7	19.6	8.0	7.8
BT, °C	37.2	36.8	36.9	36.5	36.3	36.7	36.7	36.6	36.6	37.3	35.7	37.1	37.1	36.5	36.8	36.3	36.3
Urine volume, mL/day	618	244	239	607	554	528	363	241	744	816	544	625	603	543	484	207	501
WBC, 10^3^/µL	8.8	3.5	9.5	7.3	10.5	9.0	8.6	11.2	16.9	17.3	16.3	20.2	20.8	21.9	22.1	19.4	18.6
**VCM dose**																	
165 mg/day every 8 h (37.7 mg/kg/day)			○	○	○	○											
80 mg/day every 12 h (18.2 mg/kg/day)								○									
40 mg/day every 24 h (9.1 mg/kg/day)												○	○	○	○	○	○
VCM concentration, μg/mL						27.1	18.6		36.1	22.4							
**ECMO**		○	○	○	○	○	○	○	○	○	○	○					
Rotational frequency, rpm		2010	2010	2010	2010	2010	1430	1770	1770	1640	1770	1570					
Blood volume, L/min		0.5	0.5	0.5	0.5	0.4	0.2	0.2	0.2	0.1	0.3	0.1					
Oxygen flow rate, L/min		0.4	0.3	0.3	0.1	0.2	0.1	0.3	0.2	0.1	0.1	0.1					

ALT: alanine aminotransferase, AST: aspartate aminotransferase, BT: body temperature, BUN: blood urea nitrogen, CRP: C-reactive protein, ECMO: extracorporeal membrane oxygenation, GFR: glomerular filtration rate, Hb: hemoglobin, PCT: procalcitonin, SCr: serum creatinine, VCM: vancomycin, and WBC: white blood cell.

## Data Availability

The data that support the findings of this manuscript are available upon request from the corresponding author, H.K.

## References

[1] Wilhelm M.P. (1991). Vancomycin. Mayo Clin. Proc..

[2] De Hoog M., Mouton J.W., van den Anker J.N. (2004). Vancomycin: Pharmacokinetics and administration regimens in neonates. Clin. Pharmacokinet..

[3] U.S. FDA (1998). Guidance for Industry: General Considerations for Pediatric Pharmacokinetic Studies for Drugs and Biological Products.

[4] Gijsen M., Vlasselaers D., Spriet I., Allegaert K. (2021). Pharmacokinetics of Antibiotics in Pediatric Intensive Care: Fostering Variability to Attain Precision Medicine. Antibiotics.

[5] Fletcher K., Chapman R., Keene S. (2018). An overview of medical ECMO for neonates. Semin. Perinatol..

[6] Buck M.L. (1998). Vancomycin pharmacokinetics in neonates receiving extracorporeal membrane oxygenation. Pharmacotherapy.

[7] Hahn J., Choi J.H., Chang M.J. (2017). Pharmacokinetic changes of antibiotic, antiviral, antituberculosis and antifungal agents during extracorporeal membrane oxygenation in critically ill adult patients. J. Clin. Pharm. Ther..

[8] An S.H., Lee E.M., Kim J.Y., Gwak H.S. (2020). Vancomycin pharmacokinetics in critically ill neonates receiving extracorporeal membrane oxygenation. Eur. J. Hosp. Pharm..

[9] Moffett B.S., Morris J., Galati M., Munoz F., Arikan A.A. (2018). Population Pharmacokinetics of Vancomycin in Pediatric Extracorporeal Membrane Oxygenation. Pediatr. Crit. Care Med..

[10] Schwartz G.J., Haycock G.B., Edelmann C.M., Spitzer A. (1976). A simple estimate of glomerular filtration rate in children derived from body length and plasma creatinine. Pediatrics.

[11] Schwartz G.J., Feld L.G., Langford D.J. (1984). A simple estimate of glomerular filtration ratein full-term infants during the first year of life. J. Pediatr..

[12] Villa G., Katz N., Ronco C. (2015). Extracorporeal Membrane Oxygenation and the Kidney. Cardiorenal Med..

[13] Winter M.E. (2005). Basic Clinical Pharmacokinetics.

[14] Hoie E.B., Swigart S.A., Leuschen M.P., Willett L.D., Bolam D.L., Goodrich P.D., Bussey M.E., Nelson R.M. (1990). Vancomycin pharmacokinetics in infants undergoing extracorporeal membrane oxygenation. Clin. Pharm..

[15] Pokorná P., Šíma M., Tibboel D., Slanař O. (2021). Impact of haemolysis on vancomycin disposition in a full-term neonate treated with extracorporeal membrane oxygenation. Perfusion.

[16] Amaker R.D., DiPiro J.T., Bhatia J. (1996). Pharmacokinetics of vancomycin in critically ill infants undergoing extracorporeal membrane oxygenation. Antimicrob. Agents Chemother..

[17] Friis-Hansen B. (1971). Body composition during growth. In vivo measurements and biochemical data correlated to differential anatomical growth. Pediatrics.

[18] Smith A.H., Hardison D.C., Worden C.R., Fleming G.M., Taylor M.B. (2009). Acute renal failure during extracorporeal support in the pediatric cardiac patient. ASAIO J..

[19] Lu H., Rosenbaum S. (2014). Developmental pharmacokinetics in pediatric populations. J. Pediatr. Pharmacol. Ther..

[20] Donadello K., Roberts J.A., Cristallini S., Beumier M., Shekar K., Jacobs F., Belhaj A., Vincent J.L., de Backer D., Taccone F.S. (2014). Vancomycin population pharmacokinetics during extracorporeal membrane oxygenation therapy: A matched cohort study. Crit. Care.

[21] Young D.G. (1973). Fluid balance in paediatric surgery. Br. J. Anaesth..

[22] Sherwin J., Heath T., Watt K. (2016). Pharmacokinetics and Dosing of Anti-infective Drugs in Patients on Extracorporeal Membrane Oxygenation: A Review of the Current Literature. Clin. Ther..

[23] Bartlett R.H. (1990). Extracorporeal life support for cardiopulmonary failure. Curr. Probl. Surg..

[24] Rybak M.J., Le J., Lodise T.P., Levine D.P., Bradley J.S., Liu C., Mueller B.A., Pai M.P., Wong-Beringer A., Rotschafer J.C. (2020). Therapeutic monitoring of vancomycin for serious methicillin-resistant Staphylococcus aureus infections: A revised consensus guideline and review by the American Society of Health-System Pharmacists, Infectious Diseases Society of America, Pediatric Infectious Diseases Society, and Society of Infectious Diseases Pharmacists. Am. J. Health Syst. Pharm..

[25] Mulubwa M., Griesel H.A., Mugabo P., Dippenaar R., van Wyk L. (2020). Assessment of Vancomycin Pharmacokinetics and Dose Regimen Optimisation in Preterm Neonates. Drugs R&D.

[26] Cohen P., Collart L., Prober C.G., Fischer A.F., Blaschke T.F. (1990). Gentamicin pharmacokinetics in neonates undergoing extracorporal membrane oxygenation. Pediatr. Infect. Dis. J..

[27] Bhatt-Mehta V., Johnson C.E., Schumacher R.E. (1992). Gentamicin pharmacokinetics in term neonates receiving extracorporeal membrane oxygenation. Pharmacotherapy.

[28] Southgate W.M., DiPiro J.T., Robertson A.F. (1989). Pharmacokinetics of gentamicin in neonates on extracorporeal membrane oxygenation. Antimicrob. Agents Chemother..

[29] Munzenberger P.J., Massoud N. (1991). Pharmacokinetics of gentamicin in neonatal patients supported with extracorporeal membrane oxygenation. ASAIO Trans..

[30] Kato H., Hagihara M., Okudaira M., Asai N., Koizumi Y., Yamagishi Y., Mikamo H. (2021). Systematic review and meta-analysis to explore optimal therapeutic range of vancomycin trough level for infected paediatric patients with Gram-positive pathogens to reduce mortality and nephrotoxicity risk. Int. J. Antimicrob. Agents..

[31] Uda K., Suwa J., Ito K., Hataya H., Horikoshi Y. (2019). Ototoxicity and Nephrotoxicity with Elevated Serum Concentrations Following Vancomycin Overdose: A Retrospective Case Series. J. Pediatr. Pharmacol. Ther..

[32] Lutsar I., Metsvaht T. (2010). Understanding pharmacokinetics/pharmacodynamics in managing neonatal sepsis. Curr. Opin. Infect. Dis..

